# Suicidal ideation among Canadian adults during the COVID-19 pandemic: the role of psychosocial factors and substance use behaviours

**DOI:** 10.1186/s12888-022-04353-9

**Published:** 2022-11-16

**Authors:** Nigatu Geda, Cindy Feng, Brice Peters

**Affiliations:** 1 Center for Population Studies, College of Development Studies, Addis Ababa University, Addis Ababa, Ethiopia; 2 Department of Community Health and Epidemiology, Faculty of Medicine, Dalhousie University, Nova Scotia, Canada

**Keywords:** Suicidal ideation, Depression, Anxiety, Mood disorder, Social isolation, Cannabis use, COVID-19

## Abstract

**Background:**

Suicide is one of the most important and increasing public health agenda around the world. Since the COVID-19 pandemic, concerns have been raised about the potential adverse impacts of the pandemic on suicide-related outcomes. The main objective of this study was to examine the association of psychosocial risk factors (mental health illnesses and social isolation) and substance use behaviors (cannabis and alcohol consumption) with suicidal ideation during the COVID-19 pandemic among Canadian adults.

**Methods:**

The study was conducted based on a total of 4005 persons 18 years of age or older, living in Canada's ten provinces. The data used in this study were collected during April 20–28, 2021, by Mental Health Research Canada. Multivariable logistic regression was used to determine the association of mental health conditions (anxiety, depression, and other mood disorder) before and since COVID-19 outbreaks, social isolation and living arrangement, as well as cannabis and alcohol consumption with suicidal ideation during COVID-19.

**Results:**

The results of adjusted logistic regression showed that the odds of suicidal ideation were 1.526 times higher (95% CI:1.082–2.152) among those who reported continued negative impacts of social isolation. The odds of suicidal ideation were also higher for those who were diagnosed as having depression before (OR = 3.136, 95% CI: 2.376–4.138) and since the COVID-19 pandemic (OR = 3.019, 95% CI:1.929–4.726) and 1.627 times higher (95% CI: 1.225–2.163) for those who were diagnosed as having anxiety before the COVID-19 pandemic. Those who reported having increased and those who were consuming cannabis during the pandemic were 1.970 (95% CI: 1.463–2.653) and 1.509 times (95% CI: 1.158–1.966) more likely to have thought of suicide than non-takers, respectively.

**Conclusion:**

Given the significant associations of psychosocial factors (mental health illnesses and social isolation) and cannabis use with suicidal ideation, more attention and support need to be given to adults who had mental health conditions before and since COVID-19, those who were negatively impacted by social isolation, and those are exposed to substance use (cannabis).

## Background

Suicide is one of the most important and increasing public health concerns around the world. In 2016, there were an estimated 800,000 suicides globally, and approximately 16 million suicide attempts [1]. Sixty percent of individuals with suicidal ideation transitioned to the first attempt within a year of onset [2]. The suicide mortality rate amounts to 1.4% of all deaths worldwide where most suicides are related to psychiatric diseases with depression, substance use disorders, and psychosis [3]. In Canada, the incidence of suicide has been increasing and becoming a major public health concern during the last few years. The most recent report from the Government of Canada indicated that approximately 11 people die by suicide each day and approximately 4,000 deaths per year [4].

Since the coronavirus disease 2019 (COVID-19) pandemic, concerns have been raised about the potential adverse impacts of the pandemic on mental health and suicide-related outcomes. The adverse effects of the pandemic on people with mental illness could be exacerbated by fear, self-isolation, and physical distancing [5]. During the COVID-19 pandemic, almost half of Canadians reported that their mental health had declined. Around one-fifth of Canadians screened positive for anxiety, depression, or post-traumatic stress disorder during Fall 2020. Many Canadian adults (13%) screened positive for generalized anxiety disorder [6]. This is evidenced by the substantial increase in demand for mental health helplines in Canada during the pandemic compared to previous years [6].

Timely identification of characteristics of the persons at high risk of suicide thought is critical to ensure early intervention and adequate provision of care. Scocco et al. [7] reported that the risk factors for lifetime suicidal ideation were female sex, younger cohort, fewer years of education, and earlier onset age of suicidal ideation. The 2018 study conducted in Canada indicated that 7% of people in the lowest income quintile plan suicide compared to 3% of people in the highest income quintile [4]. Some previous research has identified major depression as a major risk factor for suicidal thought [5, 6] which could be exacerbated by the pandemic. Furthermore, social isolation in response to stay-at-home orders may exacerbate mental health issues and, most critically, increase suicide risk [8–11]. Previous studies conducted around the world indicated that over 90% of people who died from suicide were affected by depression, alcohol abuse, or both [8, 9]. A study in China [10] found a strong association between mental illness and suicide after adjustment for sociodemographic characteristics. Based on data from the Netherlands, after adjusting for sociodemographic factors and all other mental disorders assessed in the survey, Sareen and Colleagues found that the baseline presence of any anxiety disorder was significantly associated with suicidal ideation and suicide attempts in a cross-sectional analysis [12].

Some studies have also documented the adverse effects of substance use on suicidal thoughts and/or attempts. A longitudinal cross-lagged analysis conducted on a sample of adolescents from Québec found that weekly cannabis uses at age 15 was associated with greater odds of suicidal ideation [11]. Another study reported a strong association between drug use (alcohol, nicotine, and cannabis dependence) and suicide behavior even after accounting for covariates [13]. Based on a sample of respondents from Ontario (*n* = 2335), Naji and colleagues (2020) concluded a positive association between cannabis use and suicidal ideation in patients with opioid use disorder [14]. Other studies have also confirmed significant association between cannabis use and suicide ideation [15–17].

In Canada, most studies have emphasized the role of commonly known sociodemographic factors (such as gender, income, employment, and rural/urban residence) in a specific region [18] or focused on a particular population group such as adolescents or people with mental illness [14, 15]. The empirical investigation of COVID-19-related suicide ideation in Canada based on national data is still scarce. The most recent study conducted by the Public Health Agency of Canada emphasized on limited predictors or risk factors [19]. Thus, the present study aims to assess a number of exposure variables, including psychological factors (depression, anxiety, and other mood disorders), social isolation, and substance use behaviors in association with suicidal ideation among adults in Canada during COVID-19.

## Methods

### Data sources

Mental Health Research Canada (MHRC) has conducted a comprehensive online survey to investigate how COVID-19 impacts the mental health of Canadians during COVID-19. The data used in this study was collected from Poll 6 survey during April 20–28, 2021. It is a cross-sectional online survey of 4005 persons 18 years of age or older, living in Canada's ten provinces. The questionnaire contained sociodemographic characteristics, behaviors and mental health disorders diagnosed by health professionals, i.e., depression, anxiety and post-traumatic stress disorder, suicide risk, pressure on parents, substance use, etc. [20]. The inclusion/exclusion criteria are Canadians (excluding Territories) above the age of 16. The survey excluded those who work in public relations or media. National results have been weighted by the most current census data in terms of gender, age, and region to ensure the total sample is representative of the population as a whole [21]. The data are available from MHRC (as Poll 1, 2, 3, 4, 5 and 6) on request.

### Outcome and exposure variables

The outcome variable of this study is suicidal ideation, measured based on self-reporting any thought of suicide between April 2020 and April 2021. The exposure variables of interest include psychosocial factors (i.e., physiological factors and social relational factors) and substance use behaviors (i.e., cannabis use and alcohol use). The psychological factors include depression, anxiety, and other mood disorders. For each of these factors, participants were asked “During the current COVID-19 outbreak in Canada, have you experienced anxiety disorder/depression/mood disorder—either before the COVID-19 outbreak or since it, received a diagnosis from a healthcare professional?” Social relational factors included in the study were living arrangement (i.e., living alone versus living with other people) and social isolation (i.e., being apart from others) during the COVID-19 pandemic”. Substance use behaviors include cannabis intake and alcohol consumption. For each of these factors, participants were asked are based on the questions “Has the quantity of cannabis/alcohol beverage you use/consume (in any form) in a typical week—Since the Coronavirus (COVID-19) outbreak in Canada increased, decreased, or stayed the same in your life?”.

### Control variables

Sociodemographic characteristics include: age (18 to 30; 31 to 40; 41 to 50; 51 to 60; and 61 to 101 years old), gender (male; female; another gender identity), total household income in 2020 (< $30,000; $30,000 to less than $50,000; $50,000 to less than $80,000; $80,000 to less than $100,000; $100,000 to less than $150,000; and >  = $150,000), and highest level of education completed (elementary or high school, college, technical /trade school or apprenticeship, university undergraduate degree, and university graduate/professional degree). Change in employment situation due to the pandemic was a binary response (yes/no). Additionally, since the pandemic public health restrictions across Canadian provinces were highly diverse, the province of residence was included.

### Statistical analysis

Data were analyzed using SPSS version 26. Descriptive analysis was used to examine the characteristics of the study sample. Logistic regression was used to assess the key risk factors associated with suicidal ideation among Canadians aged 18 and above during the COVID-19 pandemic, while adjusting for the other background factors. Multicollinearity among the explanatory variables was checked using Variance Inflation Factors (VIF). All variables having a *p*-value < 0.20 significance level were entered in the bivariate analysis [22]. Odds ratios with 95% confidence interval were calculated for each factor in the logistic regression model. Interactions were tested between significant variables to assess additive effects. All analyses were weighted [21] using the weighting variable provided in the data set to be representative of the study population.

## Results

Table 1 presents the percentage distribution of respondents by selected characteristics. Female respondents account for a little more than half of the respondents (51.4%). Close to 30% of the study participants were aged 60 and above, while those in early adulthood account for about 20%. About 30% of the respondents reported residing in households with either no income or less than $50,000 CAD per annum. Half of the respondents were married, and a quarter of respondents were single, and the remaining were either divorced or widowed. The majority of the respondents (about 80%) had above high school education. Those living alone account for 21%, while about 79% reported living with someone (parents, children, or others). Table 1 further indicates that about 20% of the study participants reported having been diagnosed by health professionals with anxiety and depression disorder either during or since the COVID-19 pandemic. Those who had other mood disorders accounted for about 9%. The percentages reporting increased alcohol and cannabis intake during the COVID-19 pandemic were 20% and 10%, respectively.

**Table 1:** Percentage distribution of participants’ characteristics, Canada, April 2021, *n* = 4005

Background Characteristics	Weighted %	Suicidal ideation		Missing (%)
		**No**	**Yes**	
**Gender**				0.0
Female	51.4	86.8	13.2	
Male	48.2	88.3	11.7	
Another gender identity	0.4	81.3	18.8	
**Age**				0.0
18–30	18.5	79.2	20.8	
31–40	16.8	83.8	16.2	
41–50	15.9	85.4	14.6	
51–60	19.4	89.2	10.8	
61–101	29.4	94.8	5.2	
**Household income**				
No income	0.8	82.1	17.9	8.6
Under $20,000	7.1	75.7	24.3	
$20,000—Less than $30,000	7.8	85.8	14.2	
$30,000—Less than $50,000	16.2	85.5	14.5	
$50,000—Less than $80,000	20.5	88.4	11.6	
$80,000—Less than $100,000	15.8	87.9	12.1	
$100,000 – Less than $150,000	19.6	89.2	10.8	
$150,000 or More	12.4	91.8	8.2	
**Province**				0.0
British Columbia	13.6	89.0	11.0	
Alberta	11.2	85.3	14.7	
Saskatchewan	3.0	86.8	13.2	
Manitoba	3.5	82.3	17.7	
Ontario	38.4	85.8	14.2	
Québec	23.5	91.7	8.3	
New Brunswick	2.2	86.4	13.6	
Nova Scotia	2.7	86.1	13.9	
Prince Edward Island	0.4	75.0	25.0	
Newfoundland and Labrador	1.5	90.2	9.8	
**Educational level**				0.6
Elementary School	0.9	82.4	17.6	
High School	20.2	84.4	15.6	
College	18.8	87.2	12.8	
Technical/Trade School/Apprenticeship	13.6	86.7	13.3	
University – Undergraduate Degree	30.0	88.4	11.6	
University – Graduate/Professional Degree	16.5	90.5	9.5	
**Change in employment situation**				0.0
Changed	52.6	85.6	14.4	
Not changed	47.4	89.7	10.3	
**Living arrangement**				0.0
Live alone	20.9	85.2	14.8	
Live with others	79.1	88.1	11.9	
**Social isolation (i.e., being apart from others)—During the current Coronavirus (COVID-19) outbreak**				1.3
Negative impact	67.7	85.6	14.4	
Neutral (no impact)	21.4	92.3	7.7	
Positive impact	10.8	89.0	11.0	
**Anxiety disorder—received a diagnosis from a healthcare professional**				0.9
Before the COVID-19 outbreak (before March 2020)	16.7	70.9	29.1	
Since the COVID-19 outbreak (Since March 2020)	4.7	71.1	28.9	
No	77.7	92.1	7.9	
**Depression—received a diagnosis from a healthcare professional**				1.2
Before the COVID-19 outbreak (before March 2020)	17.5	69.7	30.3	
Since the COVID-19 outbreak (Since March 2020)	4.5	65.4	34.6	
No	76.8	93.0	7.0	
**Other mood disorders—received a diagnosis from a healthcare professional**				0.9
Before the COVID-19 outbreak (before March 2020)	4.9	59.4	40.6	
Since the COVID-19 outbreak (Since March 2020)	3.7	70.0	30.0	
No	90.5	89.8	10.2	
**Intake of alcohol during COVID-19**				1.4
Increase	20.0	84.5	15.5	
Stay same or decrease	51.5	88.8	11.2	
Never drink	28.5	87.0	13.0	
**Cannabis intake during COVID-19**				1.8
Increase	9.5	72.5	27.5	
Stay same or decrease	17.1	81.8	18.2	
Never use	73.3	90.7	9.3	

The overall suicidal ideation is about 12% which varies across different socioeconomic groups. Suicidal ideation is higher among the youngest age group (20.8%) and lowest among the oldest age group (5.2%). The proportion is relatively higher for people with lower education (17.6%), those living alone (14.8%), and among those who reported increased alcohol (15.5%) and cannabis intake (27.5%). The percentage of suicidal ideation was much higher among those who reported having been diagnosed with anxiety (28.9%), depression (34.6%), and mood disorder (30%) since the pandemic (Table 1).

Prince Edward Island has the highest proportion (25%) of suicidal ideation (i.e., calculated by the reported suicidal ideation in the region by the total number of study participants in the same region). Québec, Newfoundland, and Labrador had a relatively low percentage of suicidal ideation (8.3% and 9.8%, respectively).

A bivariate logistic regression is presented in Table 2 to select potential variables for the multivariable regression analysis. All variables, except the gender of the respondent were associated with self-reported suicidal ideation (*p* < 0.05).

**Table 2:** Unadjusted Odds Ratio (OR) with 95% confidence intervals for the factors associated with suicidal ideation, Canada, April 2021, *n* = 4005

		**95%CI**		
	**Unadjusted** **OR**	**lower**	**Upper**	***p*-value**
**Sociodemographic variables**				
**Gender**				
Female	0.769	0.221	2.673	0.680
Male	0.658	0.189	2.290	0.511
Another gender identity ^RC^	1.000			
**Age**				
18–30	4.767	3.518	6.458	0.000
31–40	3.423	2.490	4.704	0.000
41–50	3.049	2.179	4.266	0.000
51–60	2.154	1.536	3.020	0.000
61-101^RC^	1.000			
**Household income**	0.856	0.812	0.903	0.000
**Educational level**				
Elementary School	1.942	0.780	4.832	0.154
High School	1.864	1.352	2.571	0.000
College	1.522	1.090	2.125	0.014
Technical/Trade School/Apprenticeship	1.578	1.108	2.250	0.012
University – Undergraduate Degree	1.326	0.972	1.810	0.075
University – Graduate/Professional Degree^RC^	1.000			
**Change in employment situation**				
Not changed	1.531	1.267	1.851	0.000
Changed ^RC^	1.000			
**Exposure variables**				
**Living arrangement**				
Live alone	1.247	1.003	1.550	0.047
Live with others ^RC^	1.000			
**Social isolation (i.e., being apart from others)—During the current Coronavirus (COVID-19)**				
Negative impact	1.336	0.977	1.827	0.069
Neutral (no impact)	0.704	0.481	1.030	0.071
Positive impact ^RC^	1.000			
**Depression—received a diagnosis from a healthcare professional**				
Before the COVID-19 outbreak (before March 2020)	5.733	4.662	7.050	0.000
Since the COVID-19 outbreak (Since March 2020)	6.634	4.771	9.224	0.000
No^RC^	1.000			
**Anxiety- received a diagnosis from a healthcare professional**				
Before the COVID-19 outbreak (before March 2020)	4.957	4.029	6.100	0.000
Since the COVID-19 outbreak (Since March 2020)	4.863	3.482	6.793	0.000
No ^RC^	1.000			
**Other mood disorders—received a diagnosis from a healthcare professional**				
Before the COVID-19 outbreak (before March 2020)	5.932	4.404	7.991	0.000
Since the COVID-19 outbreak (Since March 2020)	3.714	2.589	5.327	0.000
No^RC^	1.000			
**Intake of alcohol during COVID-19**				
Increase	1.203	0.934	1.549	0.153
Stay same or decrease	0.801	0.644	0.995	0.045
Never drink ^RC^	1.000			
**Cannabis intake during COVID-19**				
Increase	3.570	2.770	4.602	0.000
Stay same or decrease	2.255	1.797	2.829	0.000
Never use ^RC^	1.000			

Table 3 presents the multivariable logistic regression for associations between the main exposure variables and the risk of suicidal ideation. The predictive power of the final model was examined using the area under a ROC curve (AUC), which was estimated as 78.2% (95% CI: 76.0%-80.5%). No significant interaction effect among the variables was identified. The results of the final model indicate that the odds of suicidal ideation were 1.526 times higher (95% CI:1.082–2.152) among those who reported continued negative impacts of social isolation during the pandemic compared to those who reported positive impacts of social isolation. The odds of suicidal ideation were 3.136 times higher (95% CI: 2.376–4.138) for those who were diagnosed with depression before the pandemic as compared to those who were not and 3.019 times higher (95% CI:1.929–4.726) for those who reported having depression since the COVID-19 pandemic as compared to those who did not report depression since COVID-19. Similarly, the odds of suicidal ideation were 1.627 times higher (95% CI: 1.225–2.163) for those diagnosed with anxiety before the COVID-19 pandemic compared to those who had never been diagnosed with anxiety. Similar findings were observed for those who reported having other mood disorders. The odds of suicidal ideation were 1.929 times (95% CI: 1.361–2.736) higher for those diagnosed with mood disorder before the COVID-19 pandemic compared to those who had never been diagnosed with mood disorder.

**Table 3:** Adjusted Odds Ratio (AOR) with 95% confidence intervals for the factors associated with suicidal ideation, Canada, April 2021, *n* = 3897

		**95%CI**		
	**Adjusted** **OR**	**Lower**	**Upper**	***p*-value**
**Sociodemographic variable**				
**Age**				
18–30	3.327	2.391	4.629	0.000
31–40	2.335	1.651	3.304	0.000
41–50	2.325	1.623	3.332	0.000
51–60	1.591	1.113	2.274	0.011
61-101^RC^	1.000			
**Educational level**				
Elementary School	2.816	1.021	7.766	0.045
High School	1.552	1.088	2.213	0.015
College	1.369	0.951	1.970	0.091
Technical/Trade School/Apprenticeship	1.693	1.148	2.496	0.008
University – Undergraduate Degree	1.230	0.878	1.724	0.229
University – Graduate/Professional Degree^RC^	1.000			
**Exposure variables**				
**Social isolation (i.e., being apart from others)—During the current Coronavirus (COVID-19)**				
Negative impact	1.526	1.082	2.152	0.016
Neutral (no impact)	1.052	0.694	1.594	0.811
Positive impact	1.000			
**Depression—received a diagnosis from a healthcare professional**				
Before the COVID-19 outbreak (before March 2020)	3.136	2.376	4.138	0.000
Since the COVID-19 outbreak (Since March 2020)	3.019	1.929	4.726	0.000
No^RC^	1.000			
**Anxiety disorder—received a diagnosis from a healthcare professional**				
Before the COVID-19 outbreak (before March 2020)	1.627	1.225	2.163	0.001
Since the COVID-19 outbreak (Since March 2020)	1.597	0.999	2.551	0.050
No^RC^	1.000			
**Other mood disorders—received a diagnosis from a healthcare professional**				
Before the COVID-19 outbreak (before March 2020)	1.929	1.361	2.736	0.002
Since the COVID-19 outbreak (Since March 2020)	1.465	0.931	2.304	0.099
No^RC^	1.000			
**Intake of alcohol during COVID-19**				
Increase	0.961	0.707	1.305	0.798
Stay same or decrease	0.837	0.646	1.085	0.180
Never drink^RC^	1.000			
**Cannabis intake during COVID-19**				
Increase	1.970	1.463	2.653	0.000
Stay same or decrease	1.509	1.158	1.966	0.002
Never use ^RC^	1.000			

*RC* Reference category

Those who reported having increased and those who were consuming cannabis during the pandemic were 1.970 (95% CI: 1.463–2.653) and 1.509 times (95% CI: 1.158–1.966) more likely to have thought of suicide than non-takers, respectively. Respondents who reported decreasing their alcohol intake during the pandemic had a lower chance of suicidal ideation (AOR = 0.697; 95% CI: 0.543–0.895). The risk of suicidal ideation generally decreases as age increases. The odds of suicidal ideation are the highest among those with elementary level education (AOR = 2.816; 95% CI: 1.021–7.766) compared to those with higher university graduates. 

## Discussion

This study assessed the association of psychosocial factors and substance use behaviors with suicidal ideation among Canadian adults during COVID-19 pandemic. Given the increasing prevalence of suicidal tendencies and occurrences, this study could be timely and useful for increasing our understanding of the key risk factors for suicidal thoughts.

Our findings indicated that mental health (anxiety, depression, and other mood disorders) both before and since the COVID-19 is significantly associated with increased risk of suicidal ideation. The finding is consistent with other cross-sectional and longitudinal studies conducted in other parts of the world. For instance, based on data from the Netherlands (*n* = 7076), it was found that suicidal ideation and suicide attempts were significantly determined by anxiety disorder [12]. Similarly, Scocco and colleagues (2008) found that increased risk of suicidal thoughts among Italian adults was associated to mental disorder [7]. In the study of Australian adults, suicidal behavior was significantly determined by mental disorder. However, the reported associations became statistically non-significant when other background variables (such as urban–rural residence and SES) were used as control variables [23]. Louise (2018) noted that depression is strongly connected to suicidal ideation [24]. Another study reported that the severity of depression was a significant determinant of suicidality in both men and women [25]. A study on patients with opioid use disorder showed that those with more symptoms of anxiety or depression were more likely to report suicidal ideation [14].

Our study also found a strong positive association between social isolation and the likelihood of suicidal ideation. Social isolation is usually an expression of loose communication with close friends, family members, or significant others. It could denote a lack of support from others in the usual way. According to the Center for Disease Control and Prevention, social isolation can lead to loneliness in some people, while others can feel lonely without being socially isolated [26]. Both loneliness and social isolation are commonly considered immediate causes of suicidal thoughts [26]. Some studies even reported that poor social relationships (characterized by social isolation or loneliness) result in a 29% increase in the risk of heart disease, a 50% percent increased risk of dementia, and a 32% increased risk of stroke [27]. In general, the finding implies that social connections play a very important role in suicide prevention.

Increased intake of cannabis during COVID 19 was significantly associated with suicidal ideation. The analysis showed close to 1 in 10 respondents reported that their cannabis consumption increased since the start of the pandemic. For those who had mental health conditions, they may be particularly vulnerable to cannabis use as beliefs in its therapeutic potential. This could especially be detrimental to those with past and current mental health concerns who will bear a double risk of suicidal behavior. Interestingly, our study showed that even after adjusting for psychosocial variables, and other confounding variables, cannabis consumption remains significantly associated with suicidal ideation. Research has shown cannabis users can be common among persons prone to be impulsive, and among persons who engage in many types of high-risk behaviors that result in suicidal ideation [16, 28]. This result highlights the need of prevention interventions of suicidal ideation designed for not only people with mental health illness but also those cannabis users. Our findings are consistent with recent nationally representative study in US [29], which indicated that even people who used cannabis were more likely to have suicidal ideation and to plan or attempt suicide than those who did not use the drug at all. These associations remained regardless of whether someone was also experiencing depression. Notably, we did not find evidence for an association with alcohol use and suicidal ideation, which highlights the importance of exploring relationships with different substances independently.

Though the sociodemographic variables were used as control variables, it is worth mentioning that suicidal ideation significantly varied by the education and age of the respondents. Previous studies documented the significant role of a range of sociodemographic variables on suicidal behavior [19, 26, 30]. Having a lower education level is commonly reported as detrimental to suicidal attempts in previous studies [7, 30]. The earlier onset age of suicidal ideation was found to be a significant risk factor in suicidal risks among Italian adults [7].

Finally, it is worth mentioning the main strength and weaknesses of the study. The strength of this study is that data were collected from ten provinces and the data has been weighted to be representative of the study areas. As there are limited or no studies conducted addressing the association of the key exposure variables (i.e., psychosocial variables and substance use behaviors) simultaneously with suicidal ideation in the Canadian population, the findings of the present study will be useful in program planning and monitoring activities. The study did not collect data from three territories to the north, which limits its generalizability to these areas. While the present study provides ample evidence on the role of mental health disorders and social isolation in suicidal ideation during COVID-19, it is important to note that the findings could not show the temporal relationship between the main exposure variables and the outcome of interest. Also, since data collection did not consider severity measures (such as severity level of anxiety, depression, and other mental disorders), it is hard to assess the gradients of these factors on suicidal ideation. This may necessitate the collection of longitudinal data to see the temporal changes. The study solely focused on suicidal ideation reported for a defined period (< 12 months) which underestimates the magnitude as lifetime suicidal thoughts/behaviors were not considered. On top of that, this measure did not account for the severity of suicidal thinking, job loss and degree of contact with COVID. Given the above limitations, we suggest future studies to consider more robust Suicide Assessment Tool to properly gauge suicidal plans and thoughts.

## Conclusion

Our study found that the reported mental health disorders (anxiety, depression, and other mood disorders), cannabis use, and social isolation had significant association with suicidal ideation among Canadian adults during the COVID-19 pandemic. Promoting comprehensive psychosocial support (such as provision of essential clinical psychological and psychiatric interventions; periodic assessment, monitoring, and evaluation; and priority health services) for those with mental health conditions, those who had been negatively impacted by social isolation and those exposed to substance use (i.e. cannabis) would significantly prevent suicidal ideation. 

## Data Availability

The dataset used for this study is made available by Mental Health Research Canada: https://www.mhrc.ca/national-polling-covid.

## Abbreviations

AOR: Adjusted Odds Ratio
CI: Confidence Interval
MHRC: Mental Health Research Canada
OR: Odds Ratio
RC: Reference Category
SES: Socio Economic Status
VIF: Variance Inflation Factor
WHO: World Health Organization
